# Cross-species infection potential of avian influenza H13 viruses isolated from wild aquatic birds to poultry and mammals

**DOI:** 10.1080/22221751.2023.2184177

**Published:** 2023-03-13

**Authors:** Weiyang Sun, Menglin Zhao, Zhijun Yu, Yuanguo Li, Xinghai Zhang, Na Feng, Tiecheng Wang, Hongmei Wang, Hongbin He, Yongkun Zhao, Songtao Yang, Xianzhu Xia, Yuwei Gao

**Affiliations:** a Key Laboratory of Jilin Province for Zoonosis Prevention and Control, Changchun Veterinary Research Institute, Chinese Academy of Agricultural Sciences, Chang’chun, People’s Republic of China; b Poultry Institute, Shandong Academy of Agricultural Sciences, Ji’nan, People’s Republic of China; c Key Laboratory of Animal Resistant Biology of Shandong, Ruminant Disease Research Center, College of Life Science, Shandong Normal University, Ji’nan, People’s Republic of China; d Jiangsu Co-innovation Center for Prevention and Control of Important Animal Infectious Diseases and Zoonoses, Yangzhou University, Yang’zhou, People’s Republic of China

**Keywords:** Avian influenza virus, H13, wild aquatic birds, poultry, mice

## Abstract

Wild aquatic birds are the primary hosts of H13 avian influenza viruses (AIVs). Herein, we performed a genetic analysis of two H13 AIVs isolated from wild birds in China and evaluated their infection potential in poultry to further explore the potential for transmission from wild aquatic birds to poultry. Our results showed that the two strains belong to different groups, one strain (A/mallard/Dalian/DZ-137/2013; abbreviated as DZ137) belongs to Group I, whereas the other strain (A/Eurasian Curlew/Liaoning/ZH-385/2014; abbreviated as ZH385) belongs to Group III. In vitro experiments showed that both DZ137 and ZH385 can replicate efficiently in chicken embryo fibroblast cells. We found that these H13 AIVs can also efficiently replicate in mammalian cell lines, including human embryonic kidney cells and Madin-Darby canine kidney cells. In vivo experiments showed that DZ137 and ZH385 can infect 1-day-old specific pathogen-free (SPF) chickens, and that ZH385 has a higher replication ability in chickens than DZ137. Notably, only ZH385 can replicate efficiently in 10-day-old SPF chickens. However, neither DZ137 nor ZH385 can replicate well in turkeys and quails. Both DZ137 and ZH385 can replicate in 3-week-old mice. Serological surveillance of poultry showed a 4.6%-10.4% (15/328-34/328) antibody-positive rate against H13 AIVs in farm chickens. Our findings indicate that H13 AIVs have the replication ability in chickens and mice and may have a risk of crossing the host barrier from wild aquatic birds to poultry or mammals in the future.

## Introduction

Birds are the natural hosts of the avian influenza virus (AIV). AIVs can occasionally infect other animals, including pigs, canines, horses, marine mammals, and humans[1–4]. AIVs can be isolated from wild aquatic birds and can be divided into 16 subtypes of hemagglutinin (H1–H16) and 9 subtypes of neuraminidase (N1–N9). Additionally, AIVs can overcome host barriers and adapt to hosts other than wild aquatic birds via viral evolution and genetic variations. Studies have focussed on the highly pathogenic avian influenza virus (HPAIV), especially because HPAIVs pose a serious threat to animal and public health and economics. For example, H5N1 HPAIV was detected in 1997 [5] and was found to cause over 50% mortality in humans according to the reports of World Health Organisation [6]. However, few studies on low pathogenic avian influenza virus (LPAIV) exist. Notably, some LPAIV subtypes have frequently been reported to infect poultry, mammals, and humans in recent years, including H6N1, H7N9, and H9N2 LPAIVs [7–10]. Some amino acids mutations in viral proteins of these LPAIVs change the viral protein structures and increase viral adaptation to the host. Some critical molecular signatures have been studied that pose a vital influence on the viral adaption to host or pathogenicity, including PB2-E627K, PB2-D701N, HA-Q226L, and so on [11–14]. From the natural evolution course of LPAIVs, they may acquire one or several critical molecular signatures that increase the viral interspecies transmission capacity. An H3N8 LPAIV caused human infection in 2022 [15], and H1 and H6 LPAIVs can also infect humans [16–18]. Thus, LPAIVs can cross host barriers, causing animal and public health concerns.

The H13 AIV has emerged less infrequently than the other subtypes of AIVs. It has a narrower host range in wild birds than other AIV subtypes. The ecological divergence of wild birds as hosts of H13 AIV was found to rarely spillover from gulls to poultry [19]. The H13 AIV was first isolated from gulls in North America in 1977 [20]. Since the 1980s, other H13 isolates have been identified in the worldwide. Notably, the most of the strains were isolated from gull and tern populations [20,21]. There are three reports that H13N2 AIV strains can be isolated from turkeys in 1990 and 1991 [22–24]. The H13 AIVs can be divided into Eurasian and North American lineages and is present in North America, Europe, and Asia [25]. The events of intercontinental gene flow were identified by analyzing the genetic sequences of H13 AIVs [25]. Gene reassortment can occur between different subtypes of AIVs. Hemagglutinin (HA) is a key glycoprotein that determines the receptor binding capacity of the virus to birds or humans [26]. The H13 AIVs strongly bind to the avian receptor; however, some genetic variations in H13 subtypes can alter the amino acids located in the HA receptor binding regions [25]. A V186 amino acid mutation introduced into H13 AIV increases the viral binding ability to the human receptor analogue [27]. Therefore, H13 AIVs could occur in interspecies transmission through viral evolution.

We first isolated an H13N6 AIV from a mallard in China in 2013 [28] and another H13N8 AIV from a Eurasian Curlew in 2014, and we found that H13 AIVs could not infect adult chickens. Because the maturation of the immune system differs between the young chickens and adult chickens [29], the young chickens were used as animal models in virology research [30–32]. Notably, some reports have shown that influenza viruses’ virulence differed between young and adult chickens [33]. Young chickens have also been used to evaluate the protective efficacy of commercial AIV vaccines [34]. However, few studies have focused on H13 AIVs [24,25,35]. In our previous study, we reported serological evidence of the infection of chickens with the H13 AIVs in eastern China [36], indicating that H13 AIVs may infect poultry.

In a previous study, we found that two H13 AIV strains isolated from wild aquatic birds were unable to infect adult chickens [28]. However, whether they could infect young chickens remains unknown. Herein, we analysed the growth curve of H13 AIVs in chicken embryo fibroblast (CEF) cells and mammalian cells (including human primary airway epithelial cells). We selected chickens (1- and 10-day-old chickens), mice (3- and 8-week-old mice), turkeys, and quails to evaluate the infection ability of H13 AIVs in poultry or mammal hosts. Our findings suggest that H13 AIVs isolated from wild aquatic birds can replicate in poultry and mammal.

## Materials and methods

### Ethics statement and facility

The study protocol was conducted in accordance with the animal welfare guidelines established by the World Organisation for Animal Health. Furthermore, all animal housing and environment facilities satisfied the National Standard of Laboratory Animal Requirements (GB 14925-2010).

### RNA isolation, PCR amplification, sequencing, and Bayesian analysis

RNA was isolated from a 200 μL sample in Dulbecco’s modified Eagle’s medium (DMEM) using an RNeasy Mini kit (Qiagen, Germantown, MD, USA). Reverse transcription of viral RNA was performed using primers specific for the influenza virus (5′-AGCRAAAGCAGG-3′) and the sequences were cloned using PCR amplification methods. The viral gene segments were sequenced by the Beijing Genomics Institute (Beijing, China). The Lastergene sequence analysis software package (DNAStar, Madison, WI, USA) was used to analyze the DNA sequences. Bayesian analysis was performed for the HA gene segments using BEAST version 1.8.4. We employed the Hasegawa-Kishino-Yano (HKY) substitution model with four gamma categories and specified an uncorrelated lognormal relaxed clock and GMRF Bayesian skyride tree prior. The Markov chain Monte Carlo method was employed with a 50 million chain lengths to draw inferences using this model. The BEAST output was analysed using TRACER v1.4 (https://beast.bio.ed.ac.uk/tracer) with a 10% burn-in. Maximum-clade-credibility (MCC) trees with median heights were generated for each dataset using TreeAnnotator version 1.8.4 and visualized using the programme FigTree v1.4.2. Estimated tMRCA values were obtained from MCC trees with common ancestor heights.

### Cells, serum samples, and viruses

Primary human airway epithelial cells (HAECs) and primary human bronchial epithelial cells (HBECs) were purchased from Procell Life Science & Technology Co., Ltd., (Wuhan, China, CP-H016, CP-H009) and cultured in the specific medium CM-H016 or CM-H009. Madin-Darby canine kidney (MDCK) cells were cultured in DMEM supplemented with 5% foetal calf serum (FCS). Human embryonic kidney (293 T) cells and CEF cells were cultured in DMEM supplemented with 10% FCS, 2 mM glutamine, 10 mM HEPES, and 100 mg/mL streptomycin or 100 IU/mL penicillin. All cells were incubated at 37°C with 5% CO_2_.

Between 2015 and 2016, 328 serum samples were collected from different regions in China, including Shandong Province, Liaoning Province, and Qinghai Province. All serum samples originated from domestic chickens. Serum specimens were used to detect the antibody titres against H13 AIV.

The H13N6 virus A/mallard/Dalian/DZ-137/2013 (abbreviated as DZ137) was isolated from a mallard in Liaoning Province, China. The H13N8 virus A/Eurasian Curlew/Liaoning/ZH-385/2014 (ZH385) was isolated from a Eurasian Curlew in Liaoning Province, China. The H1N1 influenza virus A/Hebei/F076/2018 (F076) was isolated from Hebei Province, China [37]. The H10N4 AIV A/duck/Inner Mongolia/S201/2016(abbreviated as S201) was isolated from a duck in Inner Mongolia, China, in 2016. We propagated these isolated viruses in 10-day-old embryonated chicken eggs, after which the allantoic fluids were collected and stored at −80°C until use.

### Viral growth kinetics in cell culture

We evaluated the growth kinetics of the H13 AIV in HAECs, HBECs, CEF, 293 T, and MDCK cells. The cells were infected with the viruses at a multiplicity of infection (MOI) of 0.01 TCID_50_/cell. We cultured cells with DMEM containing 2 μg/mL TPCK-treated trypsin. The cultured cells were incubated at 37°C with 5% CO_2_. The HAECs and HBECs supported the influenza virus without exogenous trypsin. They were infected with H13 AIVs without TPCK-treated trypsin. We collected the supernatants at 12, 24, 36, 48, 60, and 72 h and determined the viral titres in MDCK cells. All experiments were performed in triplicate.

### Animals and experimental infection

#### Chickens

We chose young chickens as animal models to study the infection ability of H13 AIV in poultry. They were 1- or 10-day-old SPF white Leghorn chickens (from the Harbin Veterinary Research Institute, China). The stock viruses egg infectious dose (EID)_50_ titres of DZ137 and ZH385 were 10^7.5^ and 10^7.75^ EID_50_/mL, respectively. We included a 1-day-old chickens group and a 10-day-old chickens group. The chickens were infected intranasally with 10^6.0^ EID_50_ of DZ137 or ZH385 in a volume of 50 μL. Three chickens from each group were euthanized at 1-day post inoculation (dpi), 3, and 5 dpi. The animal experiment protocol and inoculation groups were the same as those for the 1-day-old chickens. Tissue samples from the nasal turbinate, tracheas, lungs, and colons were collected, and the viral titres were measured as described previously [28].

#### Mice

Three-week-old and eight-week-old female SPF BALB/c mice were purchased from the Experimental Animal Center of Charles River, which were inoculated with 10^6^ EID_50_ of DZ137 or ZH385 at a volume of 50 μL. The control group was inoculated with an equal volume of phosphate-buffered saline (PBS). Five mice in each group were observed, and their body weights were recorded for 14 days. On days 3 and 5, three mice from the experimental group were euthanized, and we collected nasal turbinate and lungs for viral titre detection. Blood was collected at 14 dpi to obtain the serum. Hemagglutination inhibition (HI) assays were performed to evaluate the antibody levels in the serum.

#### Turkeys

Two- to three-month-old female turkeys were divided into three groups for animal infection experiments: DZ137, ZH385 and control. The turkeys from the infection groups were inoculated with 10^6^ EID_50_ of DZ137 or ZH385 at a volume of 100 μL. The control group was inoculated with an equal volume of PBS. Three turkeys in the DZ137 group or ZH385 group were euthanized on day 1, 3, and 5 dpi, and the nasal turbinate, lungs, tracheas, colons, throat swabs, and anal swabs were collected to detect viral titres. On day 14, three turkeys from each group were euthanized and their blood samples were collected to separate the serum. Finally, the HI antibodies in turkeys’ serum were measured against inactivated DZ137 or ZH385 viruses.

#### Quails

One-month-old quails were divided into three groups: DZ137, ZH385, and control. The experimental group was inoculated with 10^6^ EID_50_ of DZ137 or ZH385 viruses at a volume of 100 μL. The control group was inoculated with 100 μL PBS. Three quails in the DZ137 or ZH385 group were euthanized on 1, 3, and 5 dpi. The samples, including the nasal turbinate, lungs, tracheas, colons, throat swabs, and anal swabs, were collected and titrated using 9- to 11-day-old chicken eggs. Three quails from each group were euthanized, and sera were on 14 dpi. HI assays were performed to evaluate the antibodies in serum.

### Virus titration in tissues and embryonated chicken eggs

The viral titres in tissues were evaluated after inoculating with H13 AIVs using embryonated chicken eggs. The tissues were homogenized with the TissueLyser (QIAGEN) in 1 mL DMEM. We centrifugated the tissues at 5 000 rpm for 10 min. We formulated a 10-fold dilution series with DMEM in a volume of 0.1 mL and inoculated 10-day-old embryonated chicken eggs. All embryonated chicken eggs were incubated at 37°C for 48 h, followed by harvesting the allantoic fluids. We used 1% chicken erythrocytes to evaluate the hemagglutinin assays. Viral titres were calculated following the Reed and Muench method [38].

Viral titres of H13 AIVs were tested in embryonated chicken eggs. The isolated strains were diluted 10-fold with PBS and then inoculated into 9- to 11-day-old chicken eggs. Each egg was inoculated with 100 µL of diluted strain and incubated at 37°C for 48 h. Finally, the fluids were detected using 1% chicken erythrocytes. The 50% infectious egg dose (EID_50_) was determined as described previously [28].

### HI assays

HI assays were performed following the handbook of the WHO Manual on Animal Influenza Diagnosis and Surveillance. Serum samples were treated with receptor destroying enzyme (RDE, Denka Seiken, Tokyo, Japan) and inactivated at 56°C for 30 min. The final serum dilution was 1:5. The test antigens were prepared with inactivated DZ137 and ZH385. The treated sera were diluted 2-fold serially in a 96-well micro-plates. The 4 HA units were added to the plates and incubated for about 30 min. The results were determined with 0.5% chicken erythrocytes. An HI titre against the virus (1:10) was considered positive.

### Receptor binding assays

The receptor binding affinity of H13 AIVs was evaluated by its ability to bind to treated chicken erythrocytes or sheep erythrocytes by the HA assays. The sheep erythrocytes have only α-2,3 glycans on the surface. When the chicken erythrocytes were treated with α-2,3-N-sialidase (Takara, Dalian, China), only α-2,6 glycans remained in the treated chicken erythrocytes. The chicken erythrocytes were treated with Vibrio cholerae neuraminidase (VCNA, Roche, San Francisco, CA, USA) to destroy the receptors. The F076, S201, DZ137, and ZH385 strains were diluted in the hemagglutinin titre of 2^7^. We used these erythrocytes in a 0.5% RBCs suspension to evaluate the receptor binding specificity.

### Statistical analyses

The data were analysed by analysis of variance (ANOVA) using GraphPad Prism version 5 (GraphPad Software, San Diego, CA, USA). We evaluated the differences using two-way ANOVA. *P*-values of <0.05, <0.01, and <0.001 were considered statistically significant.

## Results

### Genetic groups variations between DZ137 and ZH385 H13 AIVs

**Figure 1.**
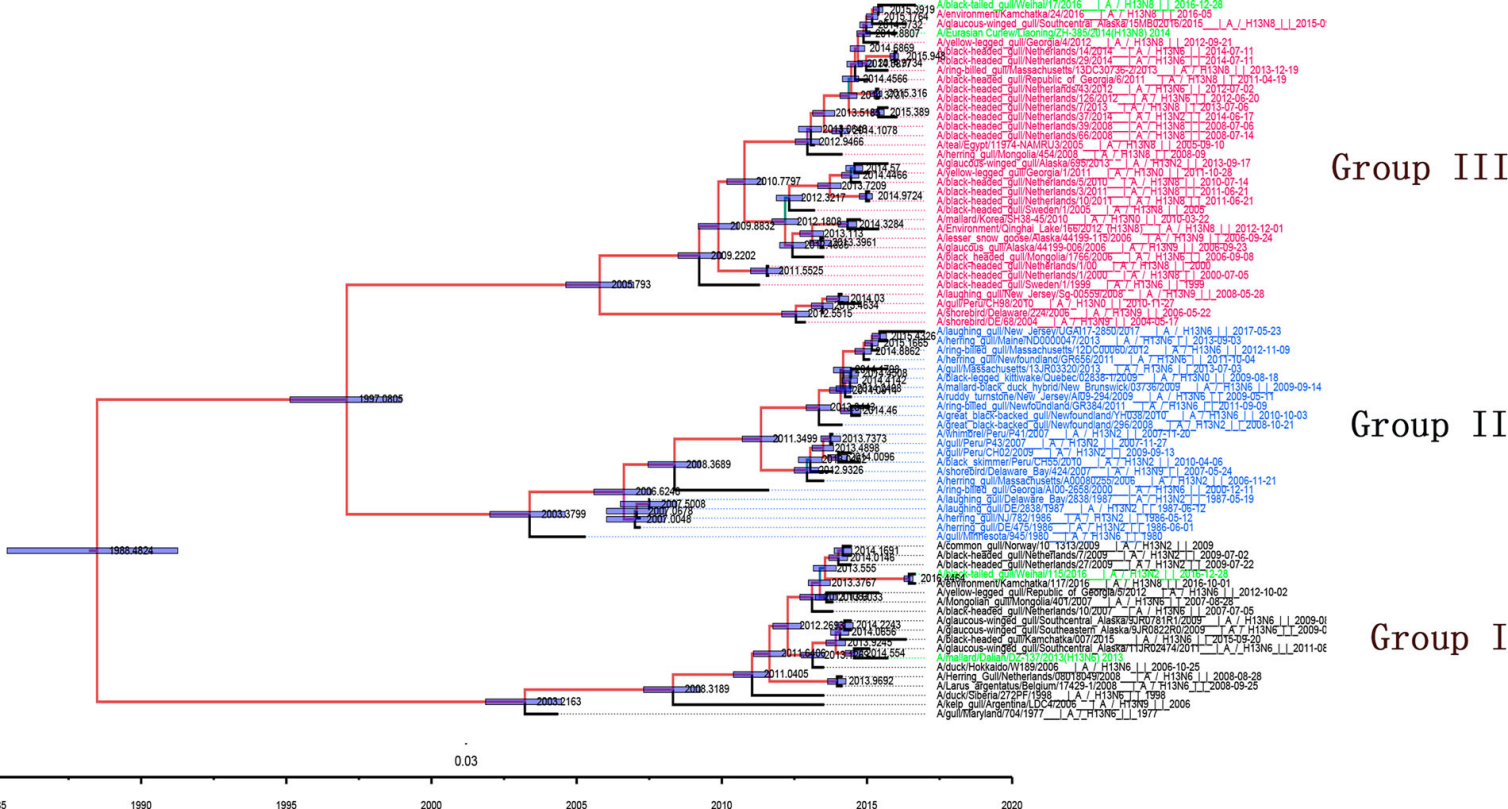
Temporally structured maximum-clade-credibility phylogenetic tree (years on the horizontal axis) of the HA gene of H13 AIVs. All H13 subtype strains were obtained from a public database. Both isolates (DZ137 and ZH385) used in this study, other isolates collected in China (A/black-tailed_gull/Weihai/115/2016 [H13N2], and A/black-tailed_gull/Weihai/17/2016 [H13N8]) are coloured green. The Bayesian posterior probabilities by which the associated taxa clustered together are shown next to the branches.

We cloned and sequenced gene segments from DZ137 and ZH385 isolated from wild aquatic birds in our laboratory. The GenBank accession numbers of the DZ137 viral gene segments are KJ907708–KJ907715, and the GenBank accession numbers corresponding to each of the eight ZH385 viral gene segments are KR010440–KR010447. Phylogenetic analysis showed that H13 AIVs could be divided into three lineages (Group I, Group II, and Group III) based on the HA gene sequences (Figure 1). Group I contains the H13 strains from Eurasia and North America. Group II comprises strains from North America and South America. Group III was mainly from Eurasia and the American continents. The genetic relationship between Group III and Group II is closer than that of Group I. The closest common ancestor between Groups III and II can be traced back to 1997, and the closest common ancestor between Group II, Group III, and Group I can be traced back to 1988. DZ137 and a Chinese H13 AIV strain (A/black tailed_gull/Weihai/115/2016) belong to Group I, whereas ZH385 and another Chinese H13 AIV strain (A/black-tailed_gull/Weihai/17/2016) belong to Group III (Figure 1).

ZH385 HA and NA come from a strain infecting a species of gulls, as is DZ137 neuraminidase. However, the DZ137 HA belongs to a duck strain. Homology analysis of the nucleotide sequences showed that the DZ137 and ZH385 gene segments shared high homology, of approximately 96.9% to 100% in GenBank (Table S1). The internal genes of these two H13 subtypes were related to those of different subtypes, including H4, H9, H6, and H16. Some representative H13 viruses were chosen to align and compare the antigenic variation between North American lineages and Eurasian lineages (Table S2). Based on the antigenic sites in the H3 HA structure, H13 AIVs had several amino acids differences in the Eurasian and North American lineages. The positions of amino acids related to antigenicity were identified in these two lineages (Figure S1).

**Table 1.** Chicken and wild bird serum survey for two AIV H13 subtypes from different locations in China, 2015–2016.

Year	Host species	Provinces	Positive HI titer results / no. tested	
			DZ137	ZH385
2016	Chicken	Shandong	14/189	30/189
2015	Chicken, ducks, and sentinel animals	Liaoning	0/35	0/35
2016	Chicken	Qinghai	1/104	4/104
Total samples			15/328	34/328

### Molecular signature differences between DZ137 and ZH385 H13 AIVs

The 627th and 701st amino acid positions of PB2 are glutamic acid (E), and aspartic acid (D), respectively (Table S3). The amino acid sequences in these two viruses are located at HA-226Q and HA-228S (Table S3), indicating that HA could not bind to the human receptors. NA 69-73 and NS 80-84 deletion was not detected in either of these two viruses (Table S3). D was present at position 92 in NS1 in both viruses. These results indicate that the two AIV H13 subtypes are low-pathogenicity influenza viruses. These findings lead to differences in virus characterization.

There were several amino acid differences in the internal genes. We identified differences in 17 amino acids in PB2, 13 amino acids in PB1, 4 amino acids in PA, 6 amino acids in NP, one amino acid in M1, 4 amino acids in NS1, and 4 amino acids in NS2 (Table S4). These amino acid differences between DZ137 and ZH385 require further study.

### H13 AIVs can replicate efficiently in CEF cells

**Figure 2.**
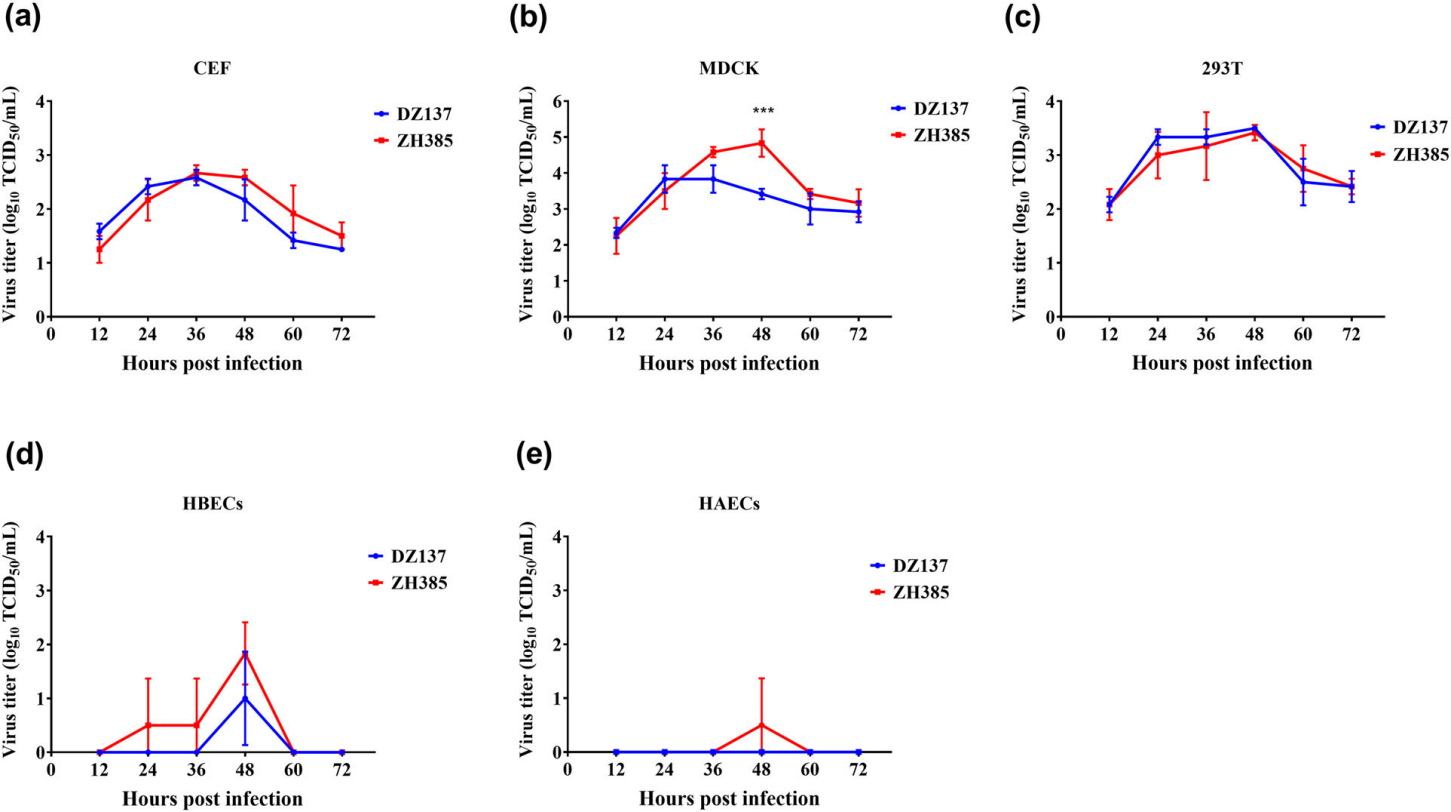
Viral growth kinetics of ZH385 and DZ137 strains in CEF, MDCK, 293 T, HBECs, and HAECs cell cultures. CEF, MDCK, 293 T, HBECs, and HAECs were infected at an MOI of 0.01. Cells were inoculated at an MOI of 0.01 TCID50/cell with DZ137 or ZH385. The supernatant of the plates was collected at 12, 24, 36, 48, 60, and 72 h post-inoculation. We determined the virus titres in MDCK cells using the TCID_50_ values. (a) CEF cells, (b) MDCK cells, (c) 293 T cells, (d) HBECs, and (e) HAECs. The virus titres are shown as the means log_10_TCID_50_/mL ± SDs. * indicates *p* < 0.05, ** indicates *p* < 0.01, and *** indicates *p* < 0.001 in comparison with the values for the DZ137 virus.

In vitro experiments, the culture supernatants were collected at different time points and measured using the 50% tissue culture infectious dose (TCID_50_) for MDCK cells. In CEF cells, both the AIV H13 subtypes reached their peak titres at 36 h. The virus titre of DZ137 reached 2.58 ± 0.14 log10 TCID_50_/mL, and the titre of ZH385 reached 2.67 ± 0.14 log10 TCID_50_/mL. After 36 h, the virus titres decreased (Figure 2a). In the MDCK cells, the virus titres were significantly different from the rest at one time point (48 h) (Figure 2b). There were no other significant differences in MDCK cells. The DZ137 strain reached a peak viral titre of 3.83 ± 0.38 log10 TCID_50_/mL at 24 h. However, the ZH385 strain reached a peak viral titre of 4.83 ± 0.38 log10 TCID_50_/mL at 48 h; the ZH385 strain reached its highest virus yield 24 h after the DZ137 strain (Figure 2b). In 293 T cells, the two H13 strains reached their peak titres at 48 h (Figure 2c). DZ137 reached 3.50 ± 0.00 log10 TCID_50_/mL, and ZH385 reached 3.42 ± 0.14 log10 TCID_50_/mL. The virus titres in CEF cells were lower than those in the 293 T cells. There was a weak replication in the HBECs (Figure 2d) and HAECs (Figure 2e). In the HBECs cells, ZH385 reached a peak viral titre of 1.83 ± 0.58 log10 TCID_50_/mL at 48 h and DZ137 reached a peak viral titre of 1.00 ± 0.87 log10 TCID_50_/mL at 48 h. The receptor binding specificity results showed that both DZ137 and ZH385 could bind to the α-2,3-linked sialic acids receptors (Figure S2), indicating that H13 AIVs can replicate efficiently in CEF cells. These H13 AIVs can also replicate efficiently in mammalian cell lines.

### Group III H13 AIV has stronger replication ability in 1-day -old chickens than Group I H13 AIV

**Figure 3.**
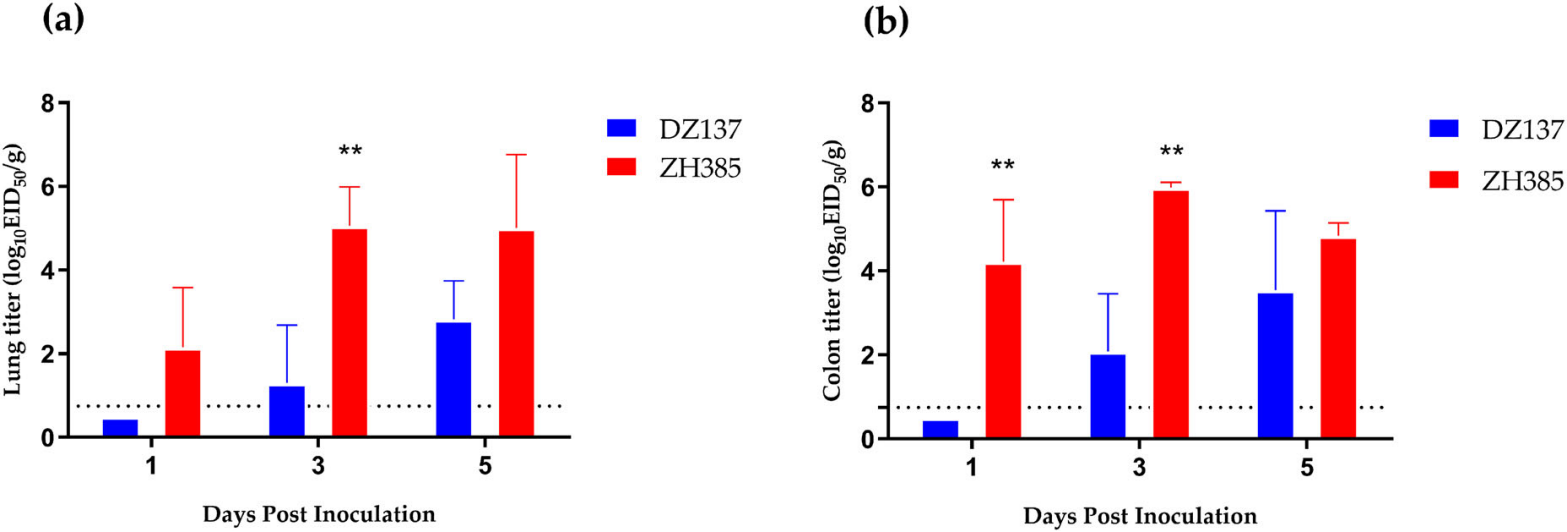
Replication of the H13 AIVs in 1-day-old chickens during the challenge experiments. Chickens were divided into two groups. Chickens (*n* = 3) and those inoculated intranasally with 50 μL of AIV H13 subtype at 10^6.0^ EID_50_. (**a**) Viral titres in lung tissues were detected at 1, 3, and 5 dpi. (**b**) Viral titres in colon tissues were detected at 1, 3, and 5 dpi. The viral titres were calculated by the Reed-Muench method. The figures show the means log_10_EID_50_/g ± standard deviations (SD). The limit of virus detection was 0.75 log_10_EID_50_/g. * indicates *p* < 0.05, ** indicates *p* < 0.01, *** indicates *p* < 0.001.

We chose 1-day-old chickens as animal models to evaluate the replication ability of two different groups (Groups I and III) H13 AIVs in poultry. We detected the lung and the colon titres at 1, 3, and 5 dpi. The ZH385 strain had a higher lung titre than the DZ137 strain (Figure 3a). At 3 dpi, we detected a titre of 5.07 ± 0.92 log_10_ EID_50_/g, which was significantly different from those of the other collection days. The DZ137 strain showed low virus titres and the virus titre at 1 dpi was not consistently determined (Figure 3a).

Similar results were obtained for the colon titres. The ZH385 strain reached 4.22 ± 1.48 log_10_ EID_50_/g at 1 dpi and 5.99 ± 0.12 log_10_ EID_50_/g at 3 dpi (Figure 3b), with significant differences. The DZ137 strain showed lower titres than the ZH385 strain. These results indicate differences in the lung and colon viral titres between the DZ137 and ZH385 strains. Overall, Group I and Group III H13 AIVs can replicate in 1-day-old chickens. Group III H13 AIVs have a stronger replication ability in chickens than Group I H13 AIVs.

### Group III H13 AIVs can replicate efficiently in 10-day-old chickens

**Figure 4.**
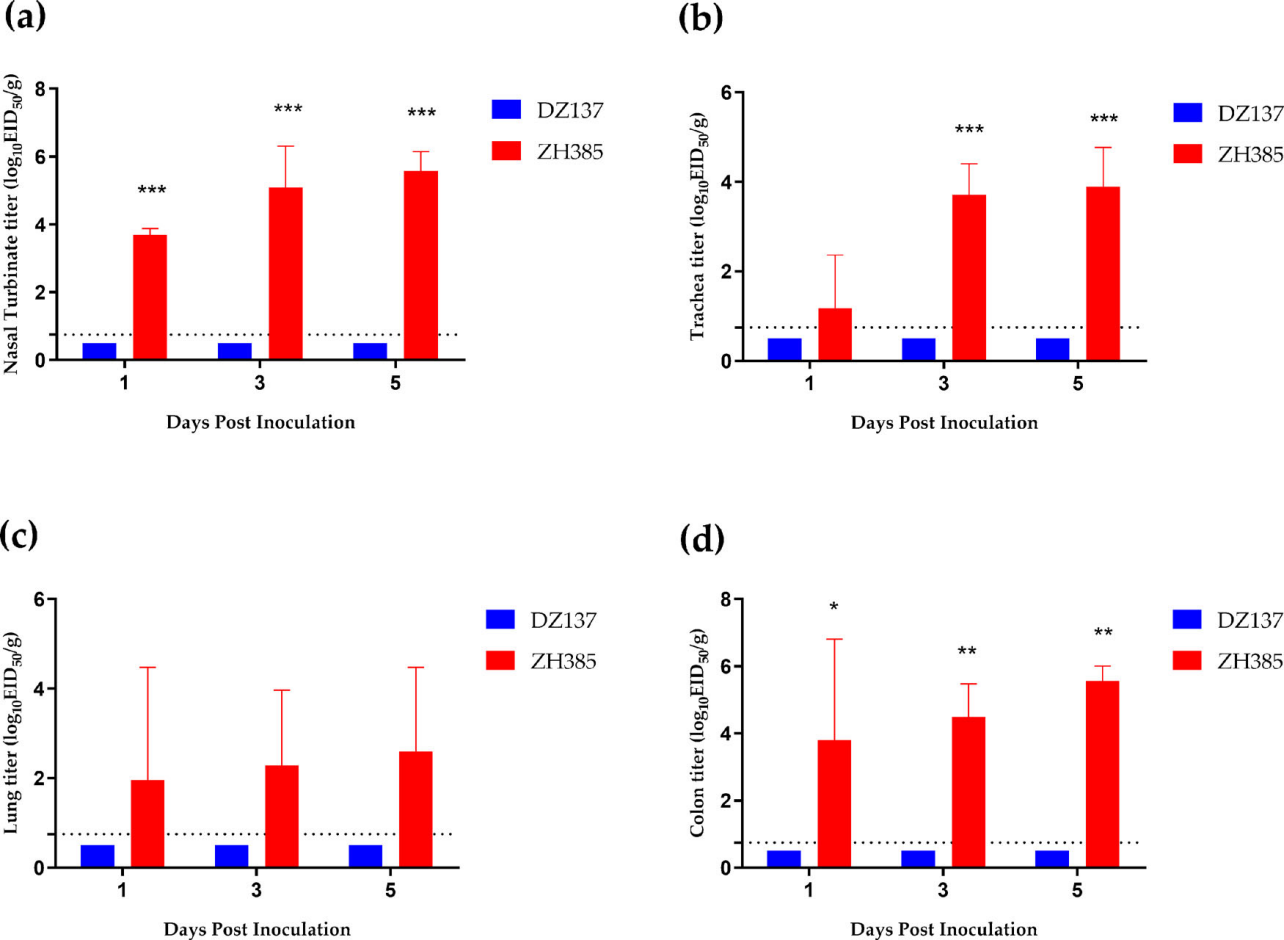
The 10-day-old chickens challenge experiment. Chickens were divided into two groups. Chickens (*n* = 3) and those inoculated intranasally with 50 μL of indicated H13 AIV at 10^6.0^ EID_50_. (**a**) Viral titres in nasal turbinate tissues were detected at 1, 3, and 5 dpi. (**b**) Viral titres in tracheal tissues were detected at 1, 3, and 5 dpi. (**c**) Viral titres in lung tissues were detected at 1, 3, and 5 dpi. (**d**) Viral titres in colon tissues were detected at 1, 3, and 5 dpi. The viral titres are shown as the means log_10_EID_50_/g ± SDs. The limit of virus detection was 0.75 log_10_EID_50_/g.

We used 10-day-old chickens to compare the replication abilities of Group I H13 and Group III H13 AIVs. DZ137 cannot infect 10-day-old chickens (Figure 4). However, ZH385 can efficiently replicate in 10-day-old chickens, and H13 viruses were detected in the nasal turbinate, trachea, lung, and colon at 1, 3, and 5 dpi (Figure 4). In the nasal turbinate tissue, we determined a significant difference in the titres at 1, 3, and 5 dpi, with viral titres of 3.70 ± 0.18 log_10_ EID_50_/g, 5.09 ± 0.22 log_10_ EID_50_/g, and 5.58 ± 0.58 log_10_ EID_50_/g, respectively (Figure 4a). In the tracheal tissue, the ZH385 strain had a titre of 3.71 ± 0.69 log_10_ EID_50_/g at 3 dpi and 3.89 ± 0.88 log_10_ EID_50_/g at 5 dpi (Figure 4b). The lung titres of the ZH385 strain had low replication rates, and there were no significant differences between the ZH385 and DZ137 strains (Figure 4c). The colon titres of ZH385 were 3.80 ± 3.01 log_10_ EID_50_/g, 4.49 ± 0.98 log_10_ EID_50_/g, and 5.56 ± 0.45 log_10_ EID_50_/g at 1, 3, and 5 dpi, respectively (Figure 4d). These results indicate that Group III H13 AIV could replicate efficiently in 10-day-old chickens.

### Group III H13 AIV has a stronger replication ability in mice compared to Group I H13 AIV

**Figure 5.**
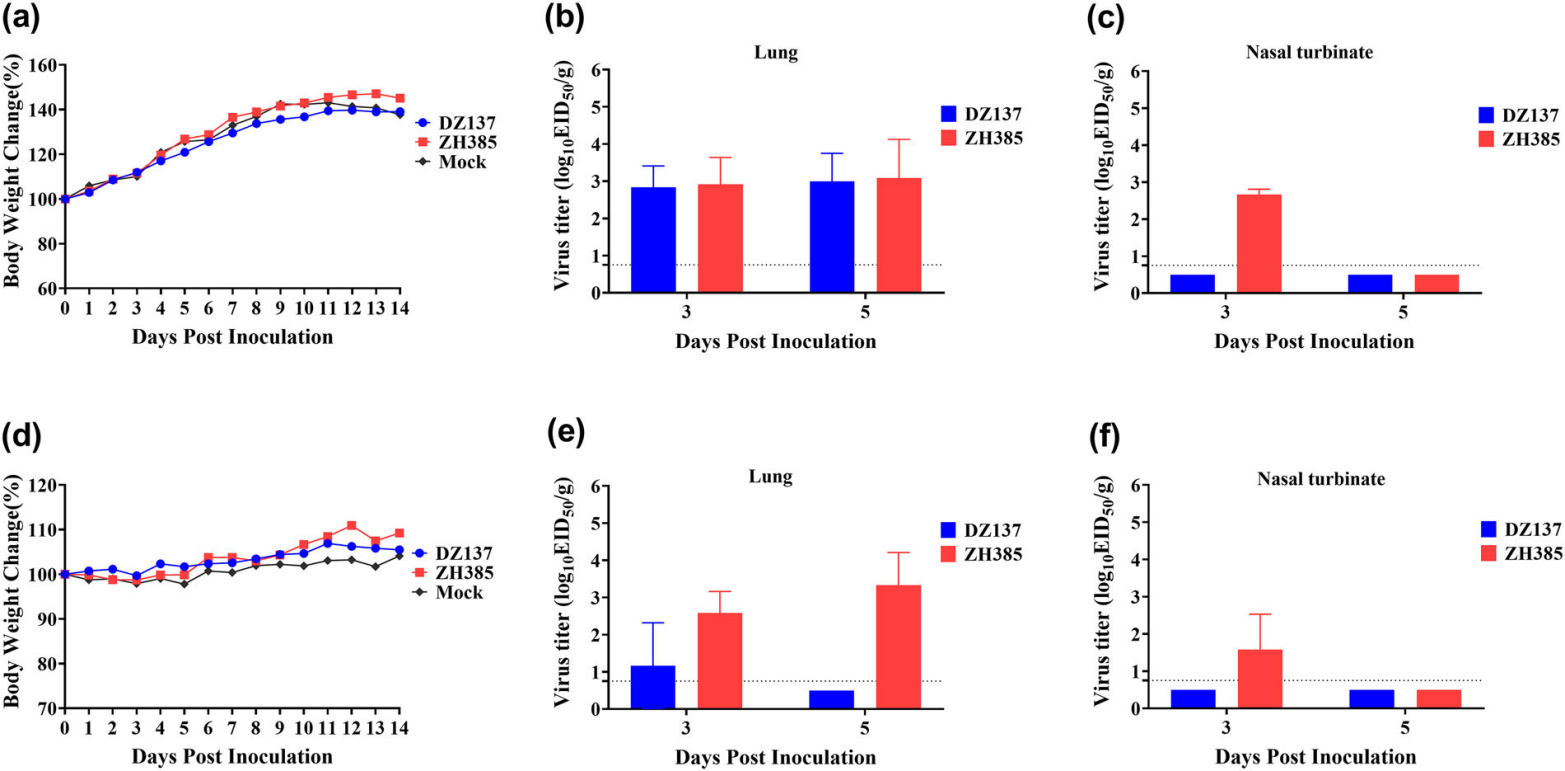
The mouse challenge experiment. Mice were divided into three groups. Mice were inoculated intranasally with 50 μL of two H13 AIVs at 10^6.0^ EID_50_. In the 3-week-old mice study, the body weight changes were recorded for 14 days, *n* = 5 (a). The virus titres were measured on day 3 and day 5 in the lungs (b) and nasal turbinate (c). In the 8-week-old mice study, the body weight changes were recorded, *n* = 5 (d). The virus replication was detected on day 3 and day 5 in the lungs (e) and nasal turbinate (f). The viral titres are shown as the means log_10_EID_50_/g ± SDs. The limit of virus detection was 0.75 log_10_EID_50_/g.

To determine the replication of H13 AIVs in mice, we selected 3- and 8-week-old mice. In the 3-week-old mice, no weight loss was observed in the DZ137, ZH385, and mock (Figure 5a). The lung viral titres of the DZ137 group were 2.83 ± 0.58 log_10_ EID_50_/g at 3 dpi and 3.00 ± 0.75 log_10_ EID_50_/g at 5 dpi (Figure 5b). The lung viral titres of the ZH385 group were 2.92 ± 0.72 log_10_ EID_50_/g at 3 dpi and 3.08 ± 1.04 log_10_ EID_50_/g at 5 dpi (Figure 5b). The nasal turbinate viral titres of the ZH385 group were 2.67 ± 0.14 log_10_ EID_50_/g at 3 dpi. No virus titres were observed at 5 dpi (Figure 5c). No virus titres of nasal turbinate were detected in the DZ137 group (Figure 5c). In the 8-week-old mice, the body weights of all three groups were not severely decreased (Figure 5d). The virus titres in the lungs of the DZ137 group were 1.17 ± 1.15 log_10_ EID_50_/g at 3 dpi (Figure 5e). The virus titres in the lungs of the ZH385 group were 2.58 ± 0.56 log_10_ EID_50_/g at 3 dpi and 3.33 ± 0.88 log_10_ EID_50_/g at 5 dpi (Figure 5e). We detected a low virus titre of 1.58 ± 0.95 log_10_ EID_50_/g in the nasal turbinate of the ZH385 group at 3 dpi and no virus titres in the nasal turbinate of the DZ137 group (Figure 5f). When inactivated DZ137 viruses were used as the test antigen, the HI antibodies reached 1:40 in 8-week-old mice and 1:20 in 3-week-old mice in the DZ137 group. When inactivated ZH385 viruses were used as the test antigen, the HI antibodies reached 1:40 in 8-week-old mice and 1:20 in 3-week-old mice in the ZH385 group (Figure S3).

### H13 AIV has limited replication ability in turkeys and quails

**Figure 6.**
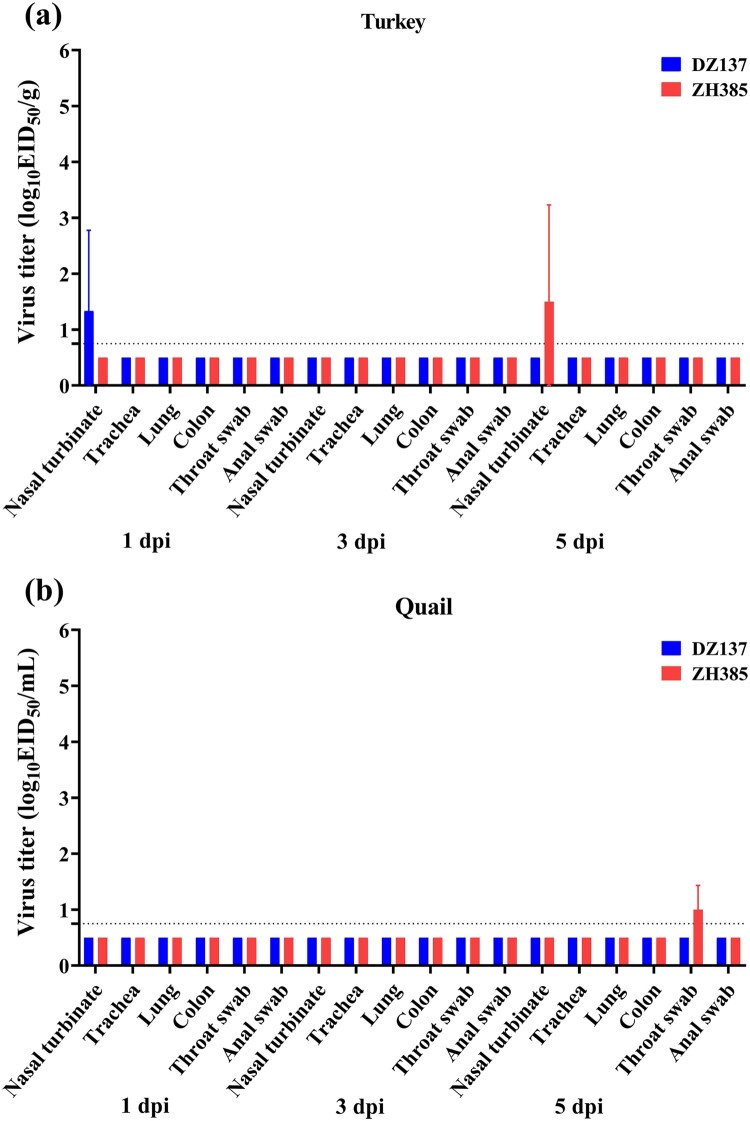
The turkeys and quails challenge experiment. The turkeys or quails were divided into two groups. Turkeys or quails (*n* = 3) were inoculated intranasally with 100 μL of indicated H13 AIV at 10^6.0^ EID_50_. The nasal turbinate, trachea, lung, colon, throat swab, and anal swab were collected and titrated at 1, 3, and 5 dpi in turkeys (a) and quails (b). The viral titres are shown as the mean log_10_EID_50_/g ± SDs or log_10_EID_50_/mL ± SDs. The limit of virus detection was 0.75 log_10_EID_50_/g or 0.75 log_10_EID_50_/mL.

To study the susceptibility of turkeys and quails, we inoculated turkeys and quails with H13 AIVs. The viral titres of the nasal turbinate in turkeys were 1.33 ± 1.44 log_10_ EID_50_/g at 1 dpi of the DZ137 group and 1.50 ± 1.73 log_10_ EID_50_/g at 5 dpi of the ZH385 group (Figure 6a). The viral titres of quail throat swabs were 1.00 ± 0.43 log_10_ EID_50_/ml at 5 dpi of the ZH385 group (Figure 6b). No viral titers were detected in the other tissues or swabs. In the turkeys experiments, the HI antibodies reached 1:20 in the DZ137 group (Figure S4). No HI antibody titres were detected in the sera of quails (Figure S5).

### Antibody-positive rate against H13 AIVs differs in farm chickens in different provinces

According to the results of the young chicken’s challenge experiment, we speculated that H13 AIVs might occasionally infect poultry. We collected and analysed chicken serum samples in China from 2015 to 2016, including Shandong Province, Liaoning Province, and Qinghai Province. These provinces were in the migratory routes for wild birds. The poultry may have more chances to be in contact with the wild aquatic birds. Fifteen samples showed detectable HI antibody titres against DZ137, and 34 samples had detectable HI antibody titres for ZH385 in the chicken serums (Table 1). One serum sample had an HI antibody titre of 40 against DZ137 and ZH385 simultaneously (Table S5). In the serum samples from the Shandong Province, 14 samples had HI titres of 40–320 (DZ137), and 30 samples had HI titres of 40–320 (ZH385) (Table S6). Furthermore, four samples presented the HI titres to DZ137 and ZH385 simultaneously. We found 15 (4.6%) samples with HI antibody titres against DZ137 and 34 (10.4%) specimens with detectable HI antibody titres against ZH385 in all serum samples.

## Discussion

LPAIVs have garnered increasing attention owing to their evolution and cross-species transmission. Some unusual LPAIVs could cause human infections, including H1N2, H3N8, H6N1, H7N4, H9N2, and H10N8 [17,18,39,40]. H13 LPAIVs are commonly isolated from gulls. Different subtypes AIVs may lead to reassortment when they co-existed in gulls or wild ducks. H13 AIVs have been isolated in the European, Asian, or American continents. Antigenicity variation was observed in different lineages of H13 AIVs [41]. With the evolution of H13 LPAIVs, they may show the risk of the cross-species transmission in new host species.

The host species barrier involves interactions including host-virus interactions and host-host interactions [42,43]. In AIVs, hemagglutinin proteins decided whether the virus can infect birds or humans and plays an important role in host restriction. Mutations in viral polymerases, NA, and NS1 genes participate in pathogenicity and interspecies transmission. The implication of mutations in mammalian adaptation is studied in AIVs. The E627K mutations in PB2 proteins are studied in most subtypes AIVs [12,44–46]. Herein, the molecular markers of host adaptation (such as E627K and D701N) were not detected in these two H13 AIV subtypes. Infection experiments in mice supported these results and indicated that the two H13 AIVs were probably not have fully adapted to mammalian hosts. No E627K or D701N mutations were observed found in other H13 subtypes strains based on the GISAID data. The sialic acid receptor types were another key factor that participates in the cross-species transmission in hosts. The results of receptor binding assays showed that the two H13 AIVs have not obtained the binding ability to human-like receptors. However, H13N8 AIV showed a dual receptor characteristic [35]. Therefore, the ecology of H13 AIVs should be monitored and attention should be paid to the risks of cross-species transmission.

Genetic reassortments may occur when the host is infected with two or more AIVs. Although one subtype of AIV may not infect other hosts directly, the exchanged genes may provide internal genes to another subtype of AIVs. The novel AIVs may pose a potential risk to animal and public health [47]. The matrix gene of H1N1 contributes to the transmission of swine influenza viruses [48]. More events of intercontinental gene exchanged were also observed in H13 AIVs [25,49,50]. PB1 gene of H13N8 AIVs may be derived from the Anseriformes virus and presented the interspecies reassortments [35]. The H13 AIVs in different groups have undergone gene flow between Eurasian and North American strains [25]. There was an interesting phenomenon that H13 AIVs isolated from the same location may belongs to different groups. Two H13N2 strains A/duck/Hokkaido/W345/2012 and A/duck/Hokkaido/WZ68/2012 were isolated in the same place and the same year [41], but A/duck/Hokkaido/W345/2012 belonged to Group III, whereas A/duck/Hokkaido/WZ68/2012 belonged to Group I. We found that DZ137 belonged to Group I, whereas ZH385 belonged to Group III. Two H13 AIVs of wild bird origin were reported in 2016 in China [36]. We found that A/black-tailed_gull/Weihai/115/2016 (H13N2) belonged to Group I, whereas both A/black-tailed gull/Weihai/17/2016 (H13N8) belonged to Group III. DZ137 and ZH385 were isolated from the same location in China in different years, and groups variation between DZ137 and ZH385 may be due to the differences in the migration routes of wild aquatic birds. With the increase in isolates of H13 AIVs, the gene flow of the H13 subtype across continents requires further investigation.

The host cell lines were useful models for studying the replication ability of the influenza virus. H13N8 AIV had a limited characteristics in MDCK or A549 cells [35]. Here, we evaluated the differences in H13 AIV growth in poultry and mammalian cells. The two H13 AIVs showed weaker replication in two different primary human airway epithelial cells than other cells. This may be due to the specificity of avian-like receptors in the two H13 AIVs. The human airway epithelial cells expressed dominantly human-like receptors with sialic acid linked to galactose through α-2,6-linkages [51]. Interestingly, two H13 AIVs showed low viral titres in primary human airway epithelial cells in this study.

In our previous study, we found that these two H13 AIV isolates could not replicate in one-month-old chickens (Figure S6), which was consistent with another study [52]. Young chickens are commonly used to investigate the efficacy and susceptibility of vaccines to AIVs [34]. Therefore, we chose young chickens as an experimental animal model to evaluate the infection ability of H13 AIVs in poultry. Group III H13 AIVs have a higher replication ability in young chickens than Group I H13 AIV. Group III H13 AIVs showed similar replication abilities in the 3- or 8-week-old mice. Both H13 AIVs belongs to Eurasian lineages but showed different infection capacities in the young chicken and young mice. This could be due to the antigenicity variation in two H13 AIVs. Most North American H13 LPAIV could infect gulls and some specific strains may infect turkeys [24]. We found that Chinese Group III H13 AIV strains have a limited replication ability in turkeys and quails in this study.

No H13 subtype AIV has been isolated from poultry until now. We investigated H13 AIV infection in chickens by serological methods in this study. By detecting the specific antibody titres in the sera of hosts, we could identify the levels of circulating influenza viruses [53,54]. We conducted a serological survey from 2015 to 2016 in China, and HI assays detected chicken serum antibody titres against H13 AIVs. Our findings indicated that chickens on the farms may have experienced the H13 AIVs infections, and there is a different antibody-positive rate in different provinces. The domestic avian sera samples were collected from provinces close to the migratory routes in China. Domestic avian may have contacted the wild birds directly or indirectly in these locations. Poultry infection with H13 strains of wild bird origin has been reported [36]. Thus, further studies are required to determine the importance of AIV monitoring in domestic poultry.

We analysed the genetic sequences of two H13 AIVs and evaluated the replication ability of H13 AIVs in poultry and mammals in vivo and in vitro. We found that Group III H13 AIVs have better replication ability in young poultry and young mice. Hence, the surveillance of H13 AIVs and the other AIVs subtypes possessing cross-species transmission risk should be increased.

## Supplementary Material

Supplemental Material
