# Supplementary material for: Cross-species infection potential of avian influenza H13 viruses isolated from wild aquatic birds to poultry and mammals
Source: Emerg Microbes Infect. 2023 Mar 13;12(1):e2184177. doi: 10.1080/22221751.2023.2184177 (PMC10013326; doi:10.1080/22221751.2023.2184177)
Supplement: Supplemental Material [file TEMI_A_2184177_SM3570.zip › TEMI2184177 Supplementary_files/A clean version of supplementary materials_Revised.doc]

Cross-species infection potential of avian influenza H13 viruses isolated from wild aquatic birds to poultry and mammals

**Table S1.** Nucleotide homology of influenza virus genes with the closest related sequences available in GenBank

| Virus | Gene | Closest related virus strain | Nucleotide identity (%) | Accession No. |
| --- | --- | --- | --- | --- |
| DZ137 | PB2 | A/velvet scoter/Mongolia/883V/2009 (H4N6) | 96.9% | KC986346.1 |
|  | PB1 | A/duck/Hokkaido/K04/2014 (H9N2) | 97.6% | LC042041.1 |
|  | PA | A/mallard/Tumuji/TMJ-748/2013(H6N2) | 100% | [KJ907678.1](https://www.ncbi.nlm.nih.gov/nucleotide/KJ907678.1?report=genbank&log$=nuclalign&blast_rank=1&RID=9G3S9WR0014) |
|  | HA | A/duck/Hokkaido/WZ68/2012 (H13N2) | 98.4% | AB812744.1 |
|  | NP | A/glaucous-winged gull/ Alaska/ 414/ 2013(H13N2) | 98.9% | [KY131041.1](https://www.ncbi.nlm.nih.gov/nucleotide/KY131041.1?report=genbank&log$=nucltop&blast_rank=2&RID=9G3HJ8XJ014) |
|  | NA | A/glaucous-winged gull/Southcentral Alaska/11JR02182/2011 (mixed) | 98.0% | CY195631.1 |
|  | M | A/common gull/Altai/805/2011 (H16N3) | 99.3% | KF462332.1 |
|  | NS | A/black-headed gull/Republic of Georgia/4/2011 (H13N8) | 98.8% | CY185541.1 |
| ZH385 | PB2 | A/black-headed gull/Republic of Georgia/4/2012 (H16N3) | 98.9% | CY185592.1 |
|  | PB1 | A/yellow-legged gull/Republic of Georgia/1/2013 (H13N8) | 98.7% | CY185631.1 |
|  | PA | A/mallard/Republic of Georgia/13/2011 (H6N2) | 98.7% | CY185582.1 |
|  | HA | A/yellow-legged gull/Republic of Georgia/1/2013 (H13N8) | 98.8% | CY185625.1 |
|  | NP | A/black-headed gull/Republic of Georgia/9/2011(H13N8) | 98.1% | [CY185660.1](https://www.ncbi.nlm.nih.gov/nucleotide/CY185660.1?report=genbank&log$=nuclalign&blast_rank=2&RID=9G4HSFJ3014) |
|  | NA | A/yellow-legged gull/Republic of Georgia/2/2013 (H13N8) | 98.2% | CY185635.1 |
|  | M | A/Armenian gull/Republic of Georgia/2/2012(H13N2) | 99.4% | [CY185348.1](https://www.ncbi.nlm.nih.gov/nucleotide/CY185348.1?report=genbank&log$=nuclalign&blast_rank=2&RID=9G4D0TEE015) |
|  | NS | A/yellow-legged gull/Republic of Georgia/1/2010 (N2) | 99.3% | CY185313.1 |

**Table S2**. Representative strains of H13 AIVs using the alignment of North American and Eurasian lineages

| Viruses | Subtypes | GISAID | GenBank | Location |
| --- | --- | --- | --- | --- |
| A/gull/Maryland/704/1977 | H13N6 | EPI407971 | CY130086 | United States |
| A/gull/ND/44036/1992 | H13N6 | EPI598254 | KF612960 | United States |
| A/pilot_whale/Maine/328_HN/1984 | H13N2 | EPI129852 | M26091 | United States |
| A/turkey/MN/1012/1991 | H13N2 | EPI598241 | KF612932 | United States |
| A/laughing_gull/DE/2838/1987 | H13N2 | EPI90268 | CY005979 | United States |
| A/gull/MN/1352/1981 | H13N6 | EPI598248 | KF612944 | United States |
| A/ring-billed gull/Georgia/AI00-2658/2000 | H13N6 | EPI454537 | CY144202 | United States |
| A/ring-billed gull/AI10-1708/MN/ 2010 | H13N6 | EPI598276 | KF612953 | United States |
| A/laughing gull/AI08-0714/NJ/2008 | H13N9 | EPI598279 | KF612956 | United States |
| A/black-headed gull/Netherlands/2/2007 | H13N6 | EPI764137 | KR087579 | Netherlands |
| A/mallard/Dalian/DZ-137/2013 | H13N6 | EPI1549513 | KJ907711 | China |
| A/gull/Astrakhan/176/1986 | H13N2 | EPI181290 | EU835899 | Russian Federation |
| A/mallard/Korea/SH38-45/2010 | H13N2 | EPI383772 | JX030406 | Korea |
| A/duck/Hokkaido/W345/2012 | H13N2 | EPI1155353 | LC336769 | Japan |
| A/great black-headed gull /Astrakhan /1420/79 | H13N2 | EPI242389 | EU293858 | Russian Federation |
| A/Environment/Qinghai Lake/166/2012 | H13N8 | EPI1036535 | / | China |
| A/black-tailed gull/Weihai/17/2016 | H13N8 | EPI1223780 | MF461188 | China |
| A/Eurasian curlew/Liaoning/ZH-385/2014 | H13N8 | EPI1619661 | KR010443 | China |

**Table S3.** Amino acid signatures of the H13 AIVs used in this study

| Protein | Amino acid position | Signatures |  |
| --- | --- | --- | --- |
|  |  | H13N8 (ZH385) | |
| PB2 | 526 | K | |
|  | 627 | E | |
|  | 701 | D | |
| HA^a^ | 226 | Q | |
|  | 228 | S | |
| NA | 69-73 | No deletion | |
| NS1 | 80-84 | No deletion | |
|  | 92 | D | |

^a^ The amino acid numbering in the HA protein is based on the H3 numbering.

**Table S4.** Amino acid differences between DZ137 and ZH385

| Gene | Amino acid positions^a^ | Identity^b^ |
| --- | --- | --- |
| PB2 | T76I, T106A, C125L, I147T, D191E, K197R, V338I, M381L, I411V, S470N, V478I, T559I, V560I, M607L, A661T, T662N, A674S | 97.8% |
| PB1 | K54T, S59T, E172D, E178G, N213S, K214R, R215K, S257T, K386R, K391N, K430R, V591I, I667V | 98.2% |
| PA | G316D, E327G, R353K, M441I | 99.6% |
| NP | A27V, I33V, V67A, S247N, V408I, S480N | 98.8% |
| M1 | R95K | 99.2% |
| NS1 | T76A, A112T, N170S, T215A | 98.3% |
| NS2 | T14A, S44L, L85H, R86K | 96.7% |

^a^ DZ137 is on the left and ZH385 is on the right.

^b^ Percent identity between DZ137 and ZH385.

**Table S5.** Chicken serum antibodies against virus subtype H13N6 (DZ137) or subtype H13N8 (ZH385) in Qinghai Province

|  | HI titres against viruses | |
| --- | --- | --- |
| Sample no. | DZ137 | ZH385 |
| **xhj35^a^** | 40 | 40 |
| mhj17 | <10 | 40 |
| mhj16 | <10 | 40 |
| mhj15 | <10 | 40 |

^a^ The bold number indicate samples containing HI titres against two H13 viruses.

**Table S6.** Chicken serum antibodies against virus subtype H13N6 (DZ137) or subtype H13N8 (ZH385) in Shandong Province

|  | HI titres against viruses | |
| --- | --- | --- |
| Sample no. | DZ137 | ZH385 |
| 10 | 40 | <10 |
| 25 | 40 | <10 |
| 51 | 40 | <10 |
| 57 | 80 | <10 |
| 64 | 80 | <10 |
| 69 | 80 | <10 |
| 84 | 80 | <10 |
| 127 | 40 | <10 |
| 136 | 80 | <10 |
| 145 | 80 | <10 |
| **99^a^** | 40 | 320 |
| **122** | 160 | 160 |
| **179** | 40 | 320 |
| **185** | 320 | 320 |
| 34 | <10 | 80 |
| 75 | <10 | 160 |
| 78 | <10 | 80 |
| 90 | <10 | 160 |
| 102 | <10 | 320 |
| 103 | <10 | 320 |
| 119 | <10 | 320 |
| 121 | <10 | 80 |
| 132 | <10 | 320 |
| 169 | <10 | 160 |
| 170 | <10 | 160 |
| 171 | <10 | 80 |
| 172 | <10 | 80 |
| 173 | <10 | 40 |
| 174 | <10 | 160 |
| 175 | <10 | 160 |
| 176 | <10 | 80 |
| 177 | <10 | 80 |
| 178 | <10 | 320 |
| 180 | <10 | 160 |
| 182 | <10 | 160 |
| 183 | <10 | 80 |
| 186 | <10 | 80 |
| 187 | <10 | 160 |
| 188 | <10 | 160 |
| 189 | <10 | 80 |

^a^ The bold numbers indicate that the samples contained HI titres against two H13 viruses.

Figure S1. Alignment and comparison of the HA1 coding sequences of H13 avian influenza strains in North American and Eurasian lineages. The amino acids in the antigenic sites are shown in red colour based on H3 HA proteins[1,2]. The representative strains in North American lineages are shown in pink colour and the strains in Eurasian lineages are shown in blue colour. The references list of representative viruses is provided in Table S4.

Figure S2. The receptor binding specificity of two H13 AIVs was determined by resialylated cRBCs. The HA assays of four strains were performed as follows: cRBCs (untreated control), sRBCs (with only α-2,3-linked sialic acid receptors), α-2,6 cRBCs (treated with α-2,3-sialidase and only have α-2,6-linked sialic acid receptors), and desial-cRBCs (treated with Vibrio cholerae neuraminidase and have no receptors). Two representative strains were used as the human influenza virus (F076) and the avian influenza virus (S201).

Figure S3. The HI antibody titers in 8-week-old mice or 3-week-old mice. The test antigens were inactivated wild-type DZ137 or ZH385 viruses. (a) The 3-week-old mice. (b) The 8-week-old mice.

Figure S4. HI antibody titres in turkeys. The test antigens were inactivated wild-type DZ137 or ZH385 viruses.

Figure S5. HI antibody titres in quails. The test antigens were inactivated wild-type DZ137 or ZH385 viruses.

Figure S6. Infections of one-month-old chickens against DZ137 and ZH385. One-month-old chickens were infected with 10^6.0^ EID_50_ of H13 subtype influenza viruses in a volume of 100 μL. Three chickens from each group were euthanized on 1, 3, and 5 dpi. The samples from the nasal turbinate, tracheas, lungs, and colons were collected and measured by EID_50_ methods. The viral titres are shown as the means log_10_EID_50_/g ± SDs. The limit of virus detection was 0.75 log_10_EID_50_/g.

**References**

1. Wang ZJ, Kikutani Y, Nguyen LT, et al. H13 influenza viruses in wild birds have undergone genetic and antigenic diversification in nature. Virus Genes. 2018 Aug;54(4):543–549.

2. Wiley DC, Wilson IA, Skehel JJ. Structural identification of the antibody-binding sites of Hong Kong influenza haemagglutinin and their involvement in antigenic variation. Nature. 1981 Jan 29;289(5796):373–378.
