# Supplementary figures and images for: Cross-species infection potential of avian influenza H13 viruses isolated from wild aquatic birds to poultry and mammals
Source: Emerg Microbes Infect. 2023 Mar 13;12(1):e2184177. doi: 10.1080/22221751.2023.2184177 (PMC10013326; doi:10.1080/22221751.2023.2184177)

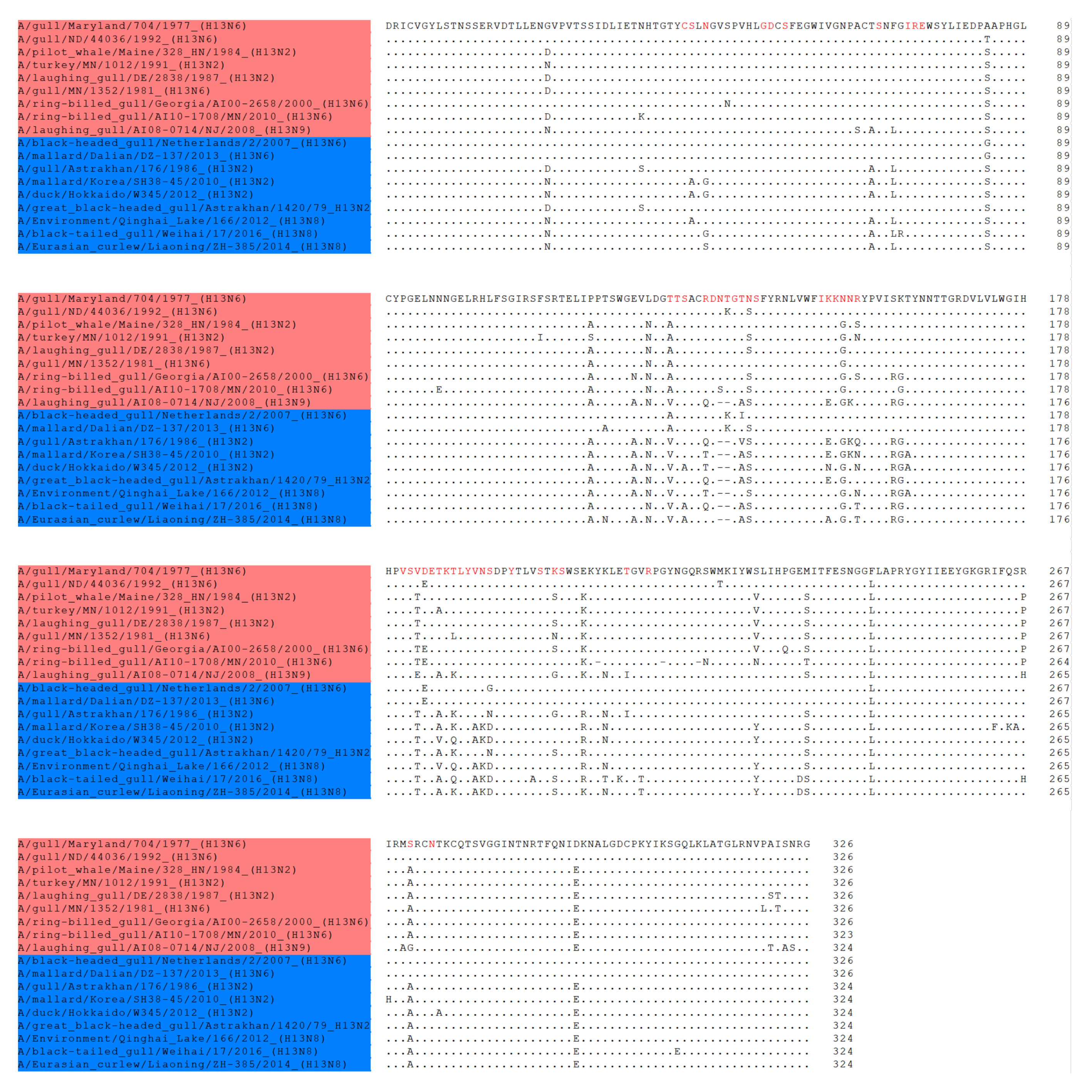

Supplement: Supplemental Material [file TEMI_A_2184177_SM3570.zip › TEMI2184177 Supplementary_files/Figure S1.tif]

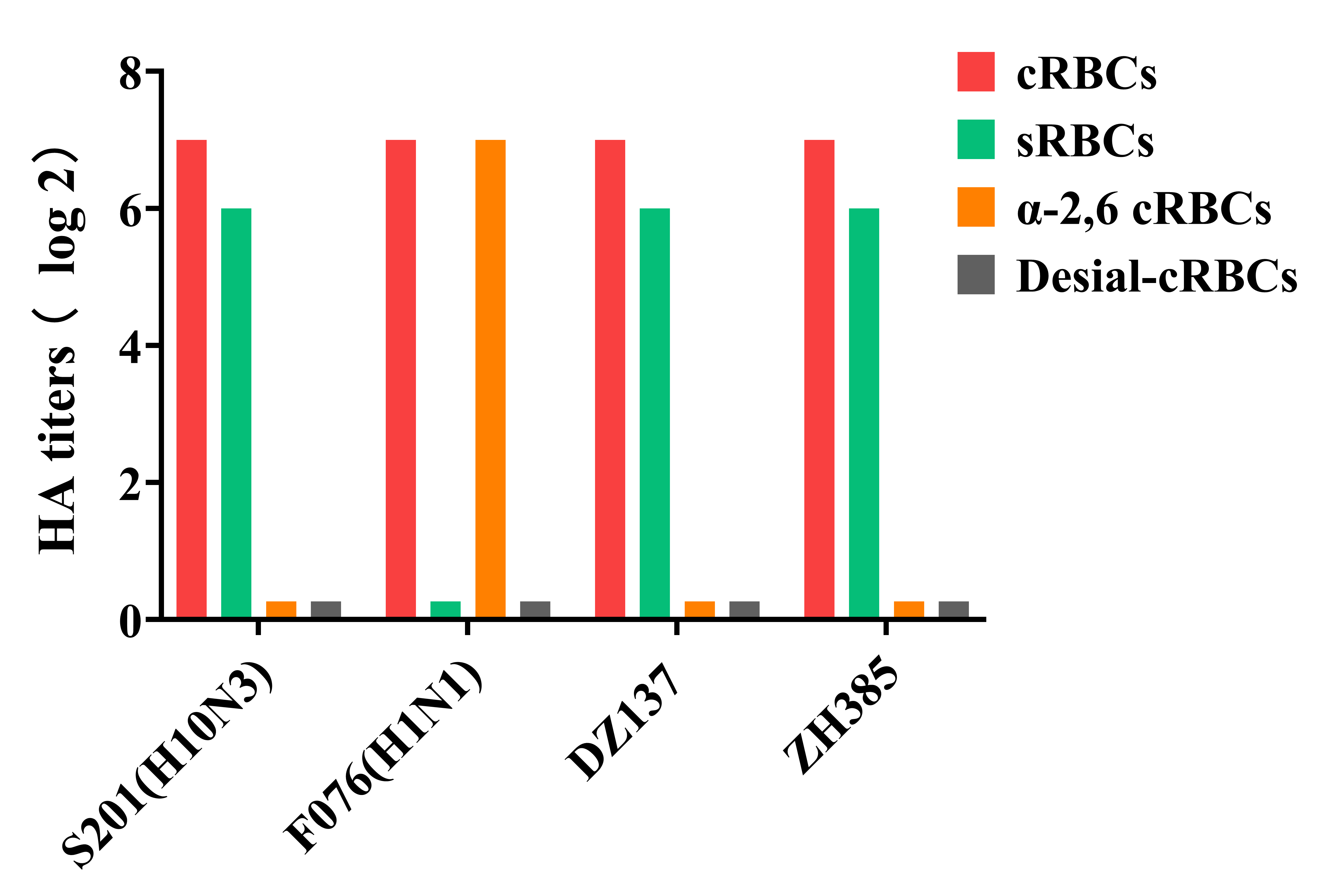

Supplement: Supplemental Material [file TEMI_A_2184177_SM3570.zip › TEMI2184177 Supplementary_files/Figure S2.tif]

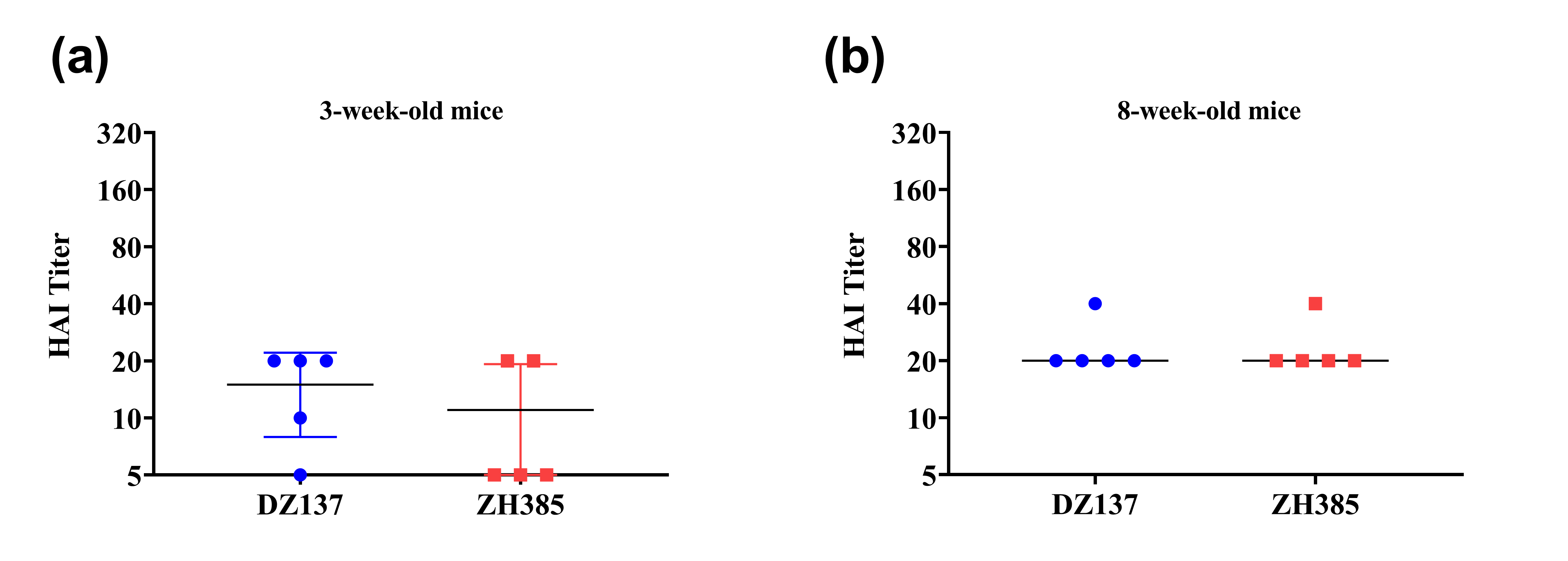

Supplement: Supplemental Material [file TEMI_A_2184177_SM3570.zip › TEMI2184177 Supplementary_files/Figure S3.tif]

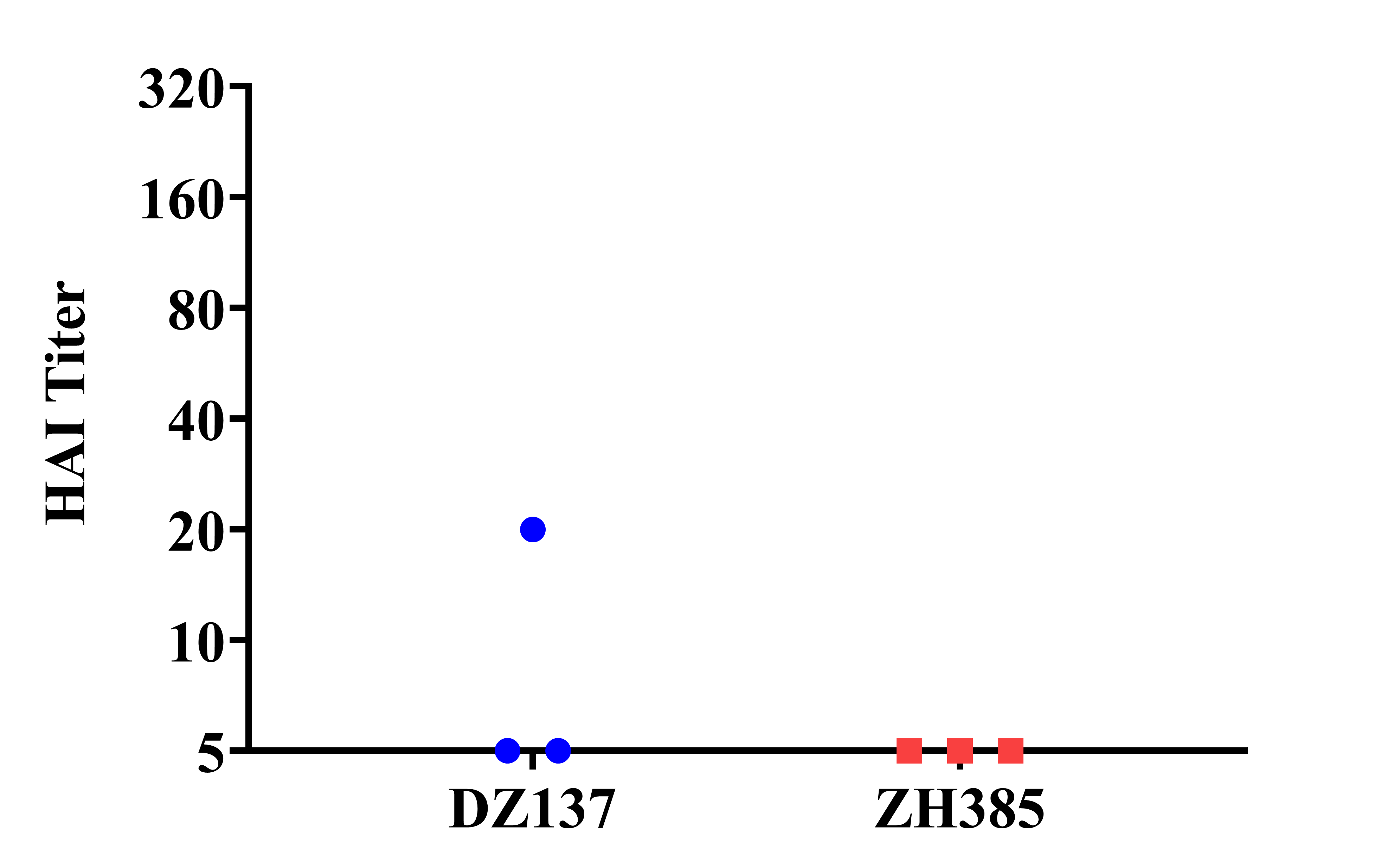

Supplement: Supplemental Material [file TEMI_A_2184177_SM3570.zip › TEMI2184177 Supplementary_files/Figure S4.tif]

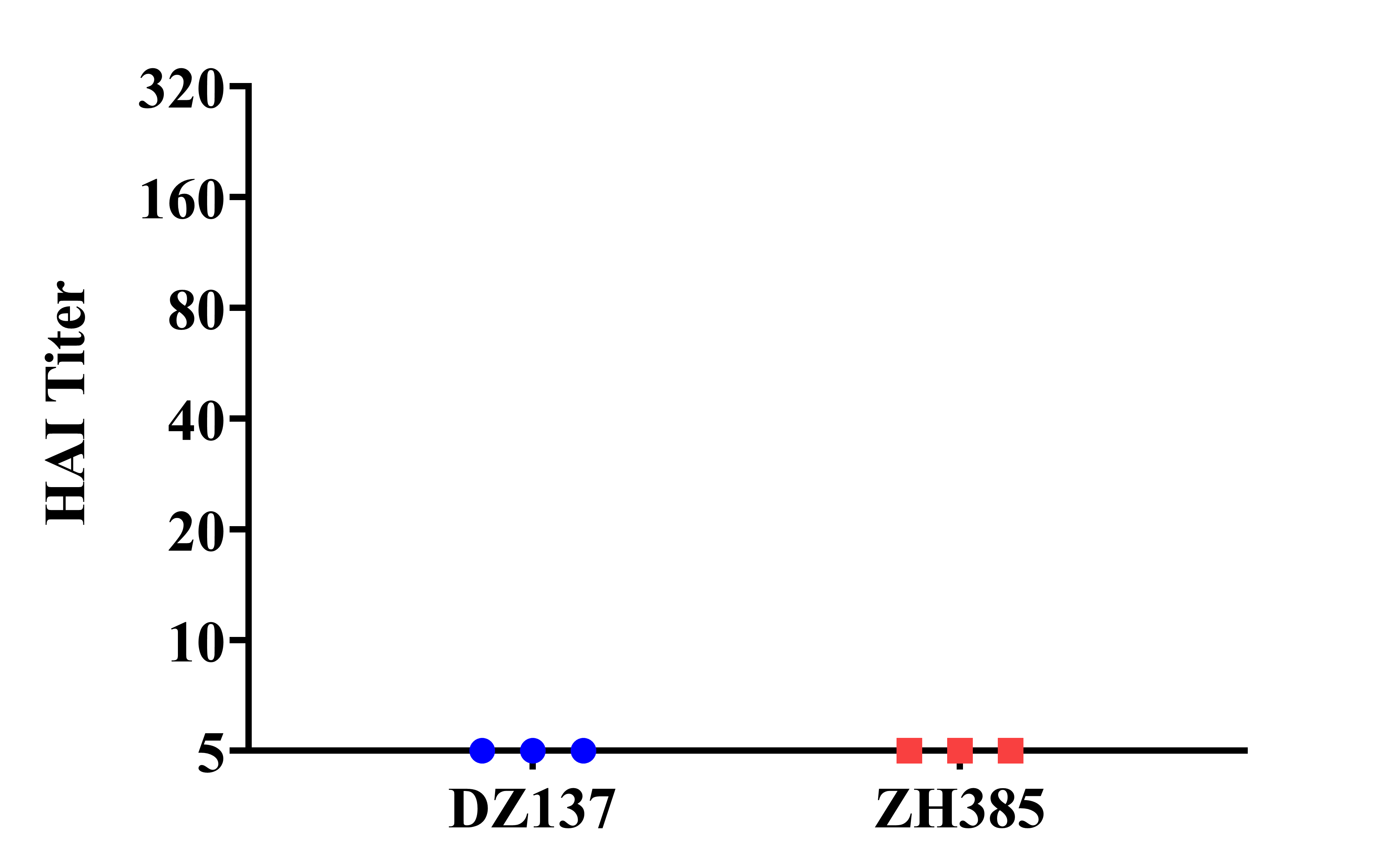

Supplement: Supplemental Material [file TEMI_A_2184177_SM3570.zip › TEMI2184177 Supplementary_files/Figure S5.tif]

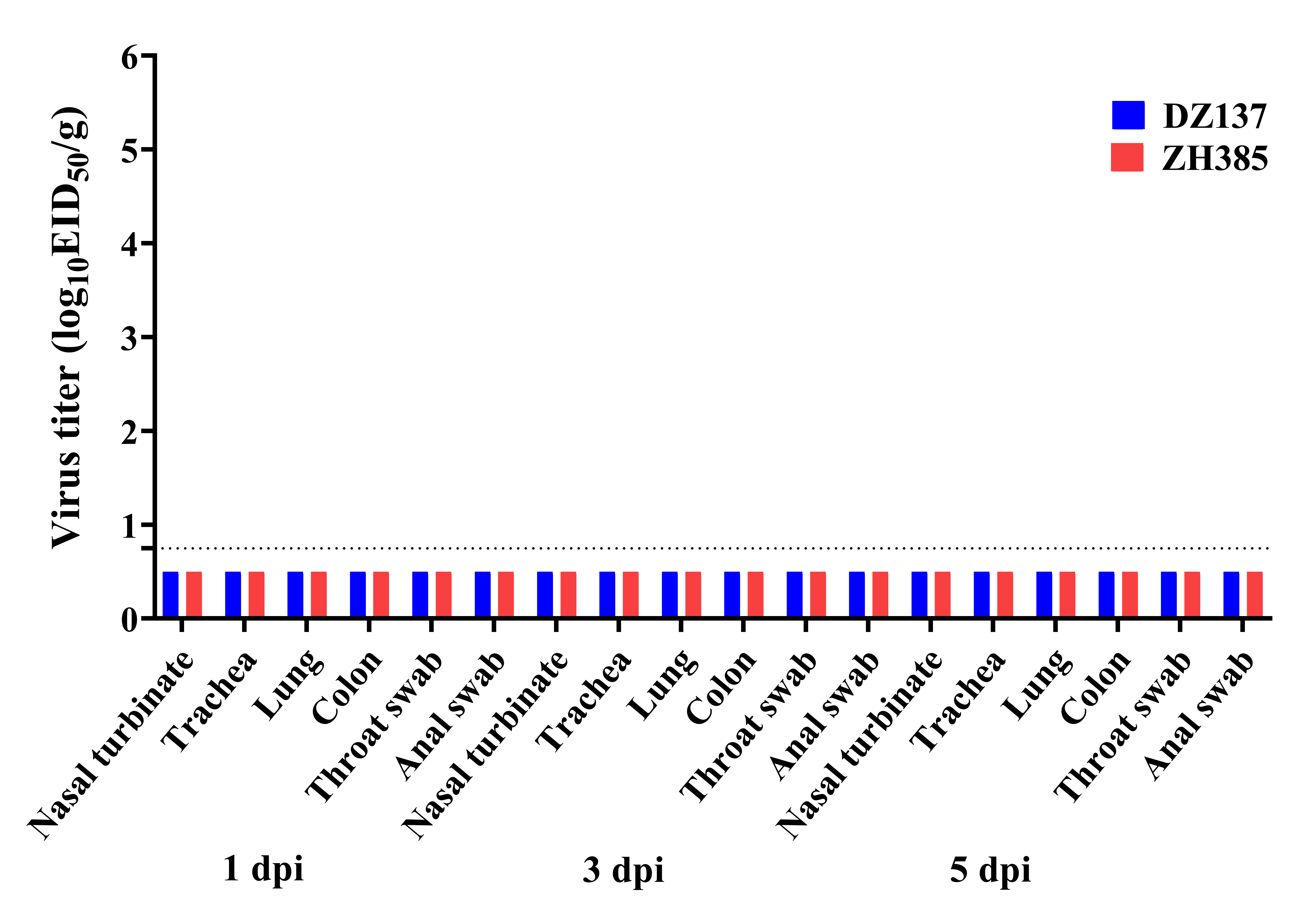

Supplement: Supplemental Material [file TEMI_A_2184177_SM3570.zip › TEMI2184177 Supplementary_files/Figure S6.tif]
